# Pneumomediastinum in Blunt Chest Trauma: A Case Report and Review of the Literature

**DOI:** 10.1155/2014/685381

**Published:** 2014-07-09

**Authors:** Gregory Mansella, Roland Bingisser, Christian H. Nickel

**Affiliations:** Department of Emergency Medicine, University Hospital Basel, Petersgraben 2, 4031 Basel, Switzerland

## Abstract

Blunt trauma is the most common mechanism of injury in patients with pneumomediastinum and may occur in up to 10% of patients with severe blunt thoracic and cervical trauma. In this case report we present a 24-year-old man with pneumomediastinum due to blunt chest trauma after jumping from a bridge into a river. He complained of persistent retrosternal pain with exacerbation during deep inspiration. Physical examination showed only a slight tenderness of the sternum and the extended Focused Assessment with Sonography for Trauma (e-FAST) was normal. Pneumomediastinum was suspected by chest X-ray and confirmed by computed tomography, which showed a lung contusion as probable cause of the pneumomediastinum due to the “Mackling effect.” Sonographic findings consistent with pneumomediastinum, like the “air gap” sign, are helpful for quick bedside diagnosis, but the diagnostic criteria are not yet as well established as for pneumothorax. This present case shows that despite minimal findings in physical examination and a normal e-FAST a pneumomediastinum is still possible in a patient with chest pain after blunt chest trauma. Therefore, pneumomediastinum should always be considered to prevent missing major aerodigestive injuries, which can be associated with a high mortality rate.

## 1. Introduction

Pneumomediastinum is defined as presence of air or other gases in the mediastinum and is also known as mediastinal emphysema. Pneumomediastinum may result from any of the following four anatomic mechanisms: first, by direct air leak from rupture of the larynx, trachea, bronchus, or esophagus into the mediastinum; second, by the “Macklin effect,” first described by Macklin in 1939 [1], who reported that a sudden increase in intrathoracic pressure results in an increased intra-alveolar pressure, leading to alveolar rupture, with air dissection along bronchovascular sheaths, and the spreading of this pulmonary interstitial emphysema into the mediastinum. The Macklin effect is involved in blunt traumatic pneumomediastinum but also in pneumomediastinum arising from various conditions, such as asthma exacerbations, positive pressure ventilation, and Valsalva maneuvers [2]; third, by perforation of a hollow abdominal viscus with subsequent dissection of air into the mediastinum via the diaphragmatic hiatus; and fourth, by air which reaches the mediastinum through and along the potential spaces and fascial planes of the neck, such as in facial trauma.

Pneumomediastinum can be categorized as atraumatic in around 20% or traumatic in around 80% of cases [3]. Traumatic pneumomediastinum is caused by blunt in around 86% or penetrating trauma in around 14% of cases [3] or by iatrogenic injury, such as that produced by mechanical ventilation or endoscopic procedures [4].

## 2. Case Presentation

A 24-year-old man presented to our emergency department complaining of persistent retrosternal pain with exacerbation during deep inspiration. The symptoms began after the patient jumped from a bridge (about 10 meters high) into a river and landed in the water on his back 6 hours prior to presentation. He was a nonsmoker and had no past history of asthma, respiratory problems, or other significant health issues. Furthermore, he denied any illicit or recreational drug use.

On exam, blood pressure was 139/89 mmHg, heart rate 88 bpm, respiratory rate 16 breaths per minute, and temperature 37.5°C. Pulse oximetry revealed an oxygen saturation of 99% on room air. The sternum was slightly tender to palpation. No subcutaneous emphysema or crepitus in the chest and neck area were observed. The lungs were bilaterally clear to auscultation. Heart sounds were regular with no murmurs, specifically no systolic crepitation. The rest of the exam was unremarkable.

**Figure 1:**
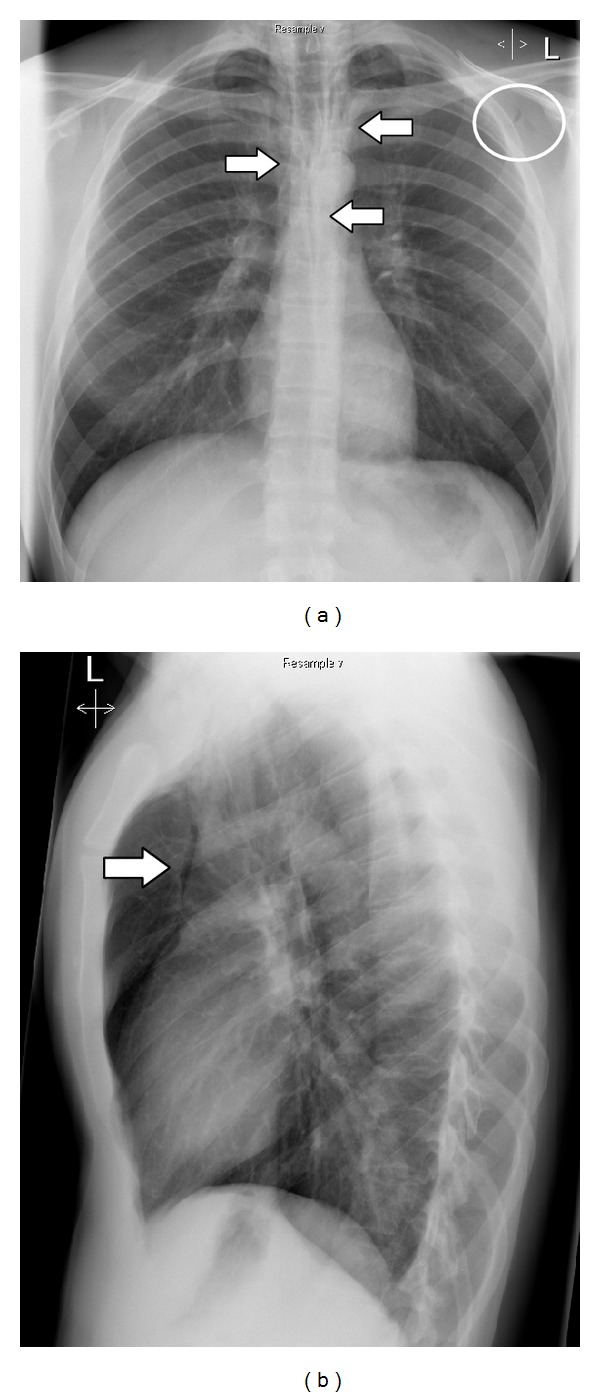
Chest X-ray showing air outlining mediastinal structures (arrows) and subcutaneous emphysema in the area of the left axilla (circle).

**Figure 2:**
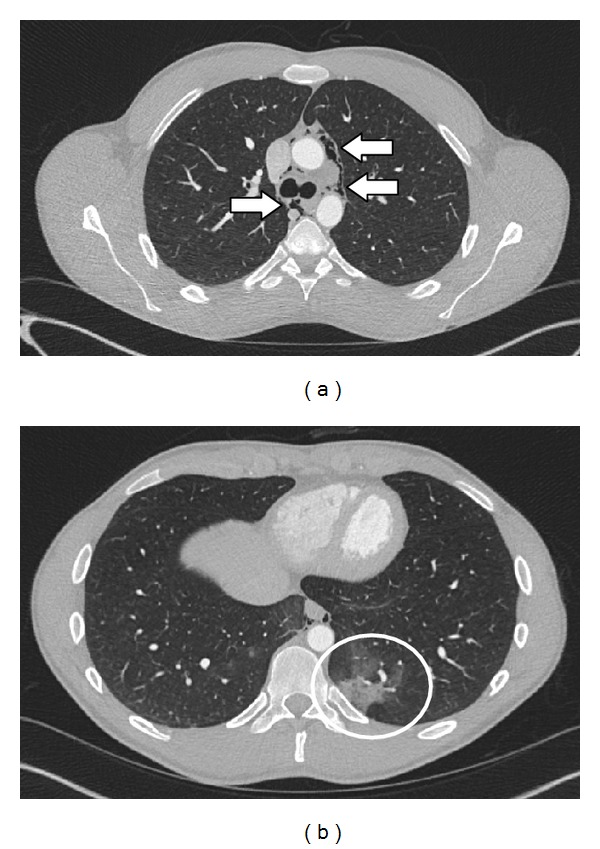
Chest computed tomography showing air outlining mediastinal structures (arrows) and a small pulmonary contusion on the left side (circle).

Laboratory exams on admission (including high sensitive cardiac troponin) were unremarkable except for a mild leukocytosis of 15,000/mm^3^ and creatine kinase of 240 U/L. An electrocardiogram showed normal sinus rhythm and no evidence of myocardial ischemia. The extended Focused Assessment with Sonography for Trauma (e-FAST) showed no evidence of a pneumothorax, pericardial/pleural effusion, or intra-abdominal fluid. The chest X-ray showed lucent streaks of gas that outlined mediastinal structures and a subcutaneous emphysema in the area of the left axilla (Figures 1(a) and 1(b)). Computed tomography confirmed the diagnosis of a pneumomediastinum and in addition showed a small pulmonary contusion on the left side (Figures 2(a) and 2(b)) without any other findings. Bronchoscopy showed no endobronchial lesion. Esophagoscopy was not performed because computed tomography with oral water-soluble contrast ruled out esophageal perforation and the patient had no gastrointestinal complaints.

**Figure 3:**
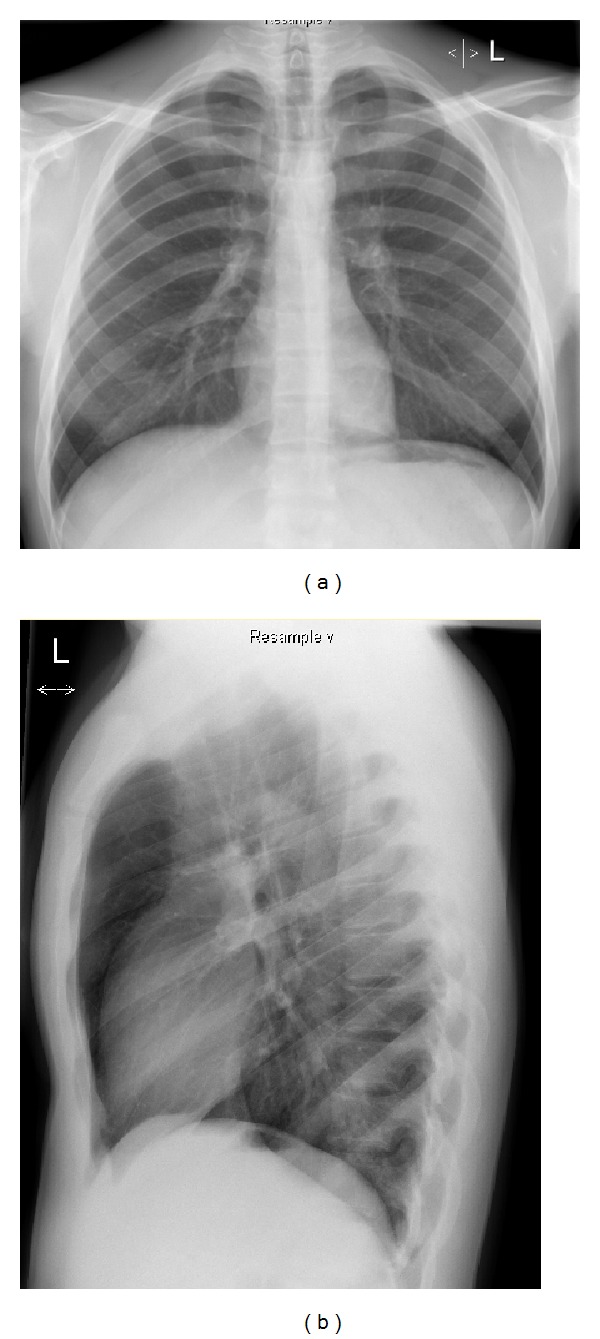
Chest X-ray showing complete resolution of findings compared to Figures 1(a) and 1(b).

The patient was managed conservatively with analgesia (acetaminophen) and a course of prophylactic antibiotics for 14 days (initially piperacillin-tazobactam intravenously, followed by amoxicillin-clavulanate orally). He was instructed to avoid maneuvers that increased pulmonary pressure (Valsalva or forced expiration). Within 4 days the pneumomediastinum resolved completely on chest X-ray (Figures 3(a) and 3(b)) and the now asymptomatic patient was discharged.

## 3. Discussion

Traumatic pneumomediastinum was first described by the pathologist Laennec in 1819 in a 4-year-old boy who was run over by a dung cart [5]. Blunt trauma is the most common mechanism of injury in patients with pneumomediastinum and may occur in up to 10% of patients with severe blunt thoracic and cervical trauma, with the highest prevalence in patients involved in high-speed motor vehicle accidents. The most common cause of traumatic pneumomediastinum results from the so-called “Macklin effect” with alveolar rupture related to primary lung trauma, positive pressure ventilation, or both [6, 7]. In a minority of cases (in up to 10% of patients), traumatic pneumomediastinum is caused by tracheobronchial ruptures or esophageal tears [8–11]. Esophageal injuries as a result of blunt trauma are a rare event, occurring in <0.1% to 1.5% of patients, and are most commonly caused by high-speed motor vehicle accidents [3, 12–15]. Traumatic injury to the tracheobronchial tree is more common than esophageal injury, occurring in up to 6% of patients [3, 11]. In contrast to esophageal injury, tracheobronchial injury is more common in blunt than penetrating trauma, with high-speed motor vehicle accidents being the most common cause [16, 17]. The mortality rate of patients who undergo surgical repair for tracheobronchial injuries can be as high as 10–25%, with a considerably high rate of complications (in up to 19% of patients) [18, 19]. Mortality rate as high as 19% has been reported for esophageal injuries [18].

The most common presenting symptom is chest pain, as in our patient, occurring in up to 75% of patients, which is typically retrosternal and pleuritic in nature and may radiate to the neck, shoulders, and arms. Other less common symptoms are dyspnoea in up to 50% and dysphagia in up to 18% of patients [3, 4].

Physical examination can be normal in up to 30% of patients with pneumomediastinum [20–22]. Signs suggestive of pneumomediastinum include subcutaneous emphysema in up to 60% of patients, which is typically detected in the neck or precordial area [4, 23] and the “Hamman's sign” (first described in 1939 by Hamman [24]) seen in up to 18% of patients [4], which is a crunching, rasping sound, synchronous with the heartbeat, heard over the precordium, and on many occasions associated with muffling of heart sounds [25]. Distended neck veins can be seen in tension pneumomediastinum if the escaped air compromises venous return. Interestingly, sternal tenderness, like in our patient, is a rare finding, being present in only 3% of patients [11]. This correlates with the likely mechanism of pneumomediastinum being due to an increase in intrathoracic pressure rather than as a result of direct mediastinal trauma.

Patients with suspected pneumomediastinum should be evaluated with frontal and lateral chest X-rays, which should include the cervical region. Radiographic signs of pneumomediastinum include lucent streaks of gas that outline mediastinal structures, elevate the mediastinal pleura, and often extend into the neck or chest wall. The lucent streak is usually seen most clearly just cranial to the heart on the left side. On lateral view, lucent streaks may outline the ascending aorta, the aortic arch, retrosternal, precardiac, periaortic, and peritracheal areas [8, 26]. Other radiographic signs of pneumomediastinum include the “continuous diaphragm sign,” which is mediastinal gas outlining the superior surface of the diaphragm and separating it from the heart [27]. Further, the “V sign of Naclerio,” which is gas outlining the lateral margin of the descending aorta and extending laterally between the parietal pleura and the medial left hemidiaphragm [28], and the “ring around the artery sign,” which is gas surrounding the mediastinal extrapericardial portion of the right pulmonary artery, can be observed [29]. Indirect radiographic evidence of pneumomediastinum includes thoracic and cervical subcutaneous emphysema, pneumopericardium, pneumoretroperitoneum, and pneumoperitoneum [8]. The radiograph should be examined for any evidence of an associated pneumothorax, which can be present in up to 84% of patients [3, 11, 30]. The presence of a pneumothorax does not necessarily indicate mediastinal organ injury in patients with pneumomediastinum [30]. In fact, Pate et al. in their retrospective study on 34 patients showed that the presence of pneumothorax was a strong negative indicator of esophageal injury and was not associated with airway injury [31]. This negative association between pneumothorax and esophageal injury may be explained by the fact that when pneumothorax is present the pneumomediastinum is more likely caused by a ruptured bleb than by esophageal injury. The presence of a pleural effusion, however, proves to be a strong predictor of esophageal injury, particularly if the patient has a history of repeated vomiting [3, 32]. In cases where chest X-rays are found to be normal (in up to 10% of patients, especially in supine radiographs) [10, 22] computed tomography may be diagnostic [33–38]. Patients at high risk for aerodigestive injury can be identified with computed tomography. Therefore, computed tomography may be used as a screening tool for aerodigestive injury in patients with pneumomediastinum to distinguish patients who can be safely observed from those in need of further evaluation. The overall sensitivity and specificity of computed tomography for major aerodigestive tract injury is 100% and 85%, respectively [11]. Computed tomography findings suggestive of a major airway injury include airway irregularity, disruption of the cartilage or tracheal wall, focal thickening or indistinctness of the trachea or main bronchi, laryngeal disruption, and concurrent pneumoperitoneum on computed tomography of the abdomen. Massive pneumomediastinum despite adequate tube drainage of pneumothoraces is also considered a suspicious finding for aerodigestive tract injury [11]. Findings on computed tomography suggestive of an esophageal injury without oral contrast include air tracking along the esophagus and pneumoperitoneum. The addition of oral contrast to computed tomography may increase the accuracy of detecting esophageal perforation and enable diagnosis of aerodigestive tract injury in patients with pneumomediastinum, with one single study [39, 40]. Some authors recommend mandatory endoscopy and/or esophagography for evaluation of all pneumomediastinum patients to exclude a major aerodigestive tract injury [41], although there is little data on the efficacy of this approach. Given the accuracy of advanced computed tomography imaging in identifying patients at high risk for aerodigestive tract injury, Dissanaike et al. conclude from their retrospective study on 136 patients that it is unnecessary to perform endoscopy and/or esophagography in the routine evaluation of blunt traumatic pneumomediastinum in the presence of a normal thoracic computed tomography [11]. Although esophageal injury has a very low incidence in blunt chest trauma but disastrous consequences if not diagnosed and treated rapidly, others may continue to prefer routine esophageal evaluation in all patients with pneumomediastinum. Another advantage of computed tomography is identifying the “Mackling effect” [42–44]. Wintermark and Schnyder showed in their retrospective study on 51 patients that the “Mackling effect” was identified in 39% of their patients with severe blunt traumatic pneumomediastinum and was associated with a significantly longer intensive care stay. No patient with the “Mackling effect” was observed beyond the age of 60 years [2]. This may result from an increased stiffness of the pulmonary interstitium in the elderly [45, 46], preventing air leak and dissection along peribronchovascular sheaths. The mere presence of the “Mackling effect” therefore does not in itself exclude a synchronous airway injury, especially in older patients. Ultrasonography of the thorax is increasingly used in the emergency department and can help in the diagnosis of pneumomediastinum, but the diagnostic criteria are not as well established as those for ruling out pneumothorax [47, 48]. Sonographic findings consistent with pneumomediastinum include the “air gap” sign, which refers to an echogenic interface anterior to the heart that obscures the view of the cardiac structures, resulting in a flickering of the heart as it alternately appears then disappears from view with each respiratory cycle. As the lung expands with inspiration, mediastinal air is displaced medially allowing the heart to be visualized with ultrasonography. As the lung contracts, the mediastinal air fills the space between the chest wall and the pleura, preventing transmission of the sonographic waves, causing the heart to momentarily disappear from view [49, 50]. The flickering of the interface varies with the respiratory cycle in patients with pneumomediastinum, which distinguishes it from the flickering with the cardiac cycle that is observed in patients with pneumothorax. This phenomenon is called the “heart point” sign [51]. The “heart point” occurs as the heart fills with blood in diastole, enlarges, and displaces the air from the pre-cardiac space, allowing the heart to transiently contact the chest wall and be visualized with ultrasonography. As the heart contracts during systole, the pneumothorax fills the space between the heart and the anterior chest wall, preventing transmission of the sonographic waves, causing the heart to momentarily disappear from view. This artefact is coordinated with cardiac systole and diastole. In our patient, pneumomediastinum was diagnosed by chest X-ray and confirmed by computed tomography, which showed an additional pulmonary contusion not visible on chest X-ray as the probable cause of the pneumomediastinum due to the “Mackling effect.” In about one-third of cases, pulmonary contusion is not evident from initial chest X-ray [52]. Pulmonary contusions evident on computed tomography, but not on chest X-ray, show better outcomes [53]. Another cause of the pneumomediastinum in our patient could have been a Valsalva maneuver he performed during the jump in the river.

Trauma patients should be treated on the basis of the overall clinical findings, associated injuries and imaging findings because pneumomediastinum alone does not seem to have major clinical significance [3]. Most patients with major aerodigestive tract injuries undergo primary repair, although good results using selective nonoperative management have been reported [17, 54–56]. Prophylactic intravenous antibiotic therapy with a broad spectrum agent should be given to all patients with major aerodigestive tract injuries due to the risk of mediastinitis. Similar to spontaneous pneumomediastinum, patients with an isolated pneumomediastinum without other injuries may be treated conservatively with analgesia, rest, and an avoidance of maneuvers that increase pulmonary pressure (Valsalva or forced expiration) [57]. Our patient with pneumomediastinum and isolated pulmonary contusion received prophylactic antibiotics as we were concerned about the development of mediastinitis, but there is no clear data recommending this approach [58]. Therapy with high concentration oxygen has been used in an effort to enhance nitrogen washout, but this is probably not necessary except in patients with severe symptoms [59, 60]. An isolated pneumomediastinum resolves within 2 to 15 days without consequences [20].

## 4. Conclusion

This present case shows that—despite minimal findings in physical examination and normal extended Focused Assessment with Sonography for Trauma (e-FAST)—pneumomediastinum should always be considered after blunt chest trauma to prevent missing major aerodigestive injuries.
